# Exploring Causes and Potential Solutions for Food Waste among Young Consumers

**DOI:** 10.3390/foods12132570

**Published:** 2023-06-30

**Authors:** Jesper Clement, Gitana Alenčikienė, Inkeri Riipi, Ugnė Starkutė, Kornelija Čepytė, Agnė Buraitytė, Aelita Zabulionė, Alvija Šalaševičienė

**Affiliations:** 1 Department of Marketing, Copenhagen Business School, 2000 Copenhagen, Denmark; 2 Food Institute of Kaunas University of Technology, 50299 Kaunas, Lithuania; gitana.alencikiene@ktu.lt (G.A.); aelita.zabulione@ktu.lt (A.Z.); alvija.salaseviciene@ktu.lt (A.Š.); 3 Natural Resources Institute Finland (Luke), 00790 Helsinki, Finland; inkeri.riipi@luke.fi; 4 Anthropos, 44253 Kaunas, Lithuania; taikomoji.antropologija@gmail.com (U.S.); taikomojiantropologija@gmail.com (K.Č.); 5 Nordic Council of Ministers Office in Lithuania, 01128 Vilnius, Lithuania; agne@norden.lt

**Keywords:** food waste, young people, food waste prevention, focus groups, interventions

## Abstract

Young consumers are often described as innovative and concerned about the environment. However, their practices sometimes are not strong enough, which are described as the attitude–behavior gap and are seen in significant amounts of food waste. The objective of this study is to focus on food waste among young consumers in high-income countries and to outline the main determinants of food waste generation. Qualitative data gathered from nine focus groups in Lithuania, Finland and Denmark (2021–2022) contribute to formulating potential intervention to decrease food waste behavior within this segment. The article provides a substantial literature review on food waste and discusses recommendations for possible interventions and further research to solve the attitude–behavior gap. The findings show four specific fields for potential solutions, related to (1) special occasions, (2) assessing food quality, (3) kitchen habits, and (4) shopping habits. Our contribution is discussed at the end of the article.

## 1. Introduction

Young consumers (Generation Z), born between 1995 and 2015, are the youngest group of consumers and are recognized as innovative and strongly influenced by social media, and as being impatient and active in online shopping [1]. However, it has also been found that these young consumers’ practices sometimes are not strong enough, described as the attitude–behavior gap [2] and exemplified as a difference between concern about the environment and a behavior that looks significantly different. Despite typical pro-environmental attitudes, young consumers in high-income countries produce significant amounts of food waste [3].

Generation Z is characterized by a low tendency to cook for themselves [4] and paired with the limited ability to either reduce or use leftovers, this appears to be one of the reasons for food waste. According to Kymäläinen et al. [5], young consumers’ food waste and unsustainable food consumption behavior stem from poorly planned or unplanned sizes of meal portions, lack of knowledge about preparing food, or unplanned events where food and meals are embedded. Many more issues that contribute to food waste among young Finnish consumers have been identified, such as too large food packaging sizes, routines, and habits when needing to change a diet, poor taste, allergies or gastric problems, social issues (e.g., gender or generation stereotypes, “old-fashioned” family values), and the economic situation [5].

This is also in line with a Danish study based on quantitative research, which shows that more than 38% of food waste comes from the plate [6], and this study finds that young people’s cooking abilities in this phase of life are among several reasons for food waste. Other studies highlight young people’s lack of motivation to plan dinners and point to the importance of focusing on in-store behavior to reduce food waste. However, a paradox exists of which young consumers with low income are very well aware: that it is unwise to lose money by throwing away edible food [7].

The objective of this study is to outline the main determinants of food waste generation and ways to decrease food waste behavior through potential intervention within this segment. The guiding research question is as follows: *How can the attitude–behavior gap among young people be minimized?* To answer this question, we conducted a systematic literature search followed by data from nine focus groups, collected between December 2021 and January 2022 in Lithuania, Finland, and Denmark.

There are several definitions of food waste and food loss across the literature, but there is no common or universal definition of food waste. In this study, we use the terms edible versus inedible food waste, as well as the terms avoidable versus unavoidable food waste. As an overall definition, we lean to the Food and Agriculture Organization of the United Nations (FAO)’s identification of food waste as losses occurring at the end of the food chain [8].

The systematic search for previous food waste interventions was carried out using the Web of Science database throughout June, July, and August 2021. The keywords included were food waste and interventions. The initial search identified 1280 results from the Web of Science Core Collection, of which 300 were in the field of Nutrition Dietetics, 267 in Environmental Sciences, 181 in Public Environmental Occupational Health, 149 in Food Science Technology, 123 in Engineering Environmental, 111 in Green Sustainable Science Technology, 75 in Environmental Studies, and 64 in Medicine General Internal. Meanwhile, in the social sciences field, the number of publications was much lower: 45 in Multidisciplinary Sciences, 33 in Agricultural Economics Policy, 29 in Economics, 22 in Business, and 20 in Behavioral Sciences. In comparison, until 2016, the number of publications per year varied from 50 to 70, this number increased to 114 from 2017 and, with continuous interest, reached 227 in 2020.

## 2. Young Consumers as More Than One Segment

Roodhuyzen et al. [9] outlined how research on food waste often has conflicting findings, and they called for a better research design to study the impact of context-specific interventions. Further, the authors underline that it might be a mistake to target all young people in the same way just because they are young. There is a risk of overseeing factors such as attitudes, social norms, and habits that may have different impacts within the same target group.

A study among Croatian university students showed that young consumers can be segmented into four groups [10]. Those concerned about economic effects of food waste (26.5%), those neglecting food waste (about 20%), well-informed consumers (about 35%), and those fully aware but not ready to take health risks (about 18%). The study was conducted among university students, and most of the students (78%) were living with their parents. Other studies have revealed that the country of origin or even the region of young consumers is a discriminant variable in the management of food, especially food waste. Bravi et al. [3] compared the behavior and attitudes of young consumers in the United Kingdom and two Mediterranean countries (Spain and Italy) and concluded that differences in the approach to food and food waste are possible based on cultural and behavioral aspects of the country itself.

Aschemann-Witzel et al. [11] studied the relationship between food-related lifestyle patterns and food waste using approximately 4200 consumers across five northern and western European countries. Researchers have identified five consumer profiles for which various food waste reduction interventions can be targeted. Based on this study, lifestyle patterns regarding food are linked to differences in food waste, choice of suboptimal food, and awareness of food waste [11]. One relevant subgroup of lifestyles among young people is the segment representing students. Regardless of age, this group has several characteristics across the types of education and study lines, and one important characteristic is their lifestyle when studying away from home. This segment has been the target of many food waste studies because they seem to change their view of food when they start to manage their own budgets and buy their own groceries [12].

Furthermore, there is a difference between living on campus and living off campus in apartments or students’ homes, where the latter segment has a more direct responsibility for food purchases, food preparation, and food storage. Some aspects of different perceptions of communication regarding food waste timing or messages have been determined in consumers with high and low mindfulness levels [13], where people with high mindfulness are more aware of and are better able to cope with needs in a here-and-now situation [14]. This insight into segmentation criteria is critical for the development of better strategies for reaching different segments of youth and assessing the effects of interventions.

## 3. Food Waste as a Social Construct

Anthropological research on food and eating emphasizes that food is a social and cultural construct [15,16] and what is classified as edible and inedible foods differs among different societies and cultures. According to Sidney Mintz [17], who is considered one of the founders of contemporary anthropology of food, it is said that “… different groups eat different foods and in different ways; all feel strongly about what they do eat and do not eat, and about the ways they do it”. Therefore, edibility is not a fixed category but rather something that is constantly reconsidered and negotiated, and when food is food or trash differs in time and space [18]. Industrialization of the food system has an important influence on this classification through a new form of food marketing [19] and by reducing the culturally embedded knowledge of food practices [18].

When edibility is not fixed or universal, one important question is when and why people classify food as edible or inedible. Blichfeldt et al. [18] showed how consumers’ enactment of edibility depends on ideologies, feelings, and skills. Participants used two strategies to decide whether food was still edible: objectification and internalization. An example of objectification is the strict use of the “best-before” information as a determining factor. The internalization strategy refers to the act of using senses or is described as common sense. The choice between the two strategies depended on the type of food that was assessed, as well as personal ideology and feeling toward food. Particularly important for the view on food waste among young people is that the internalization strategy seems to reduce food waste, whereas the objectification strategy increases food waste [18]. Providing people with skills to assess food through the senses might be an intervention to reduce food waste in households.

The literature on food and waste has shown how consumer behavior formed in discursive contexts of what was considered good food and eating, suggesting that wasteful behavior rarely depended only on individual behavior. The authors emphasized the discursive tensions between the two approaches, either as food waste and food safety [20] or as food waste and the way healthy eating is understood and portrayed in public [21]. Discourse in the media and public policy might pull home practices in conflicting directions. On the one hand, creative campaigns emphasizing the use of leftovers and by that reducing food waste and, on the other hand, conflicting with food safety are problematic in saving and reusing the very same leftovers [20].

Another tension forms around food waste and the discursive knowledge of healthy eating. Through an ethnographic example of a family’s household, Evans [21] shows that purchase choices can be made, even knowing that part of the food purchased will be wasted, for example, buying a variety of vegetables that could not be fully eaten and that will decay. However, such choices are made to meet the expectations of eating and cooking properly and healthily. These discursive tensions point to a broader context of how the food waste problem is articulated and how different realms of knowledge produce uncertainty in people’s minds, regardless of the facts. Therefore, there is a need to address these discursive issues to understand the reasons for food waste among young consumers.

### A Young Person’s Lifestyle and Household

Several studies have addressed food waste in private households and have outlined one or several issues causing waste. For an overview of the factors that might cause food waste, see the review by Roodhuyzen et al. [9], where 116 different factors were joined and related to stages within the household, such as planning, shopping, storing, preparing, serving, and consuming. In a review by Principato et al. [22], wasteful behavior is related to psychological, social, situational, demographic, and socioeconomic factors. Together, these reviews outline several reasons for food waste and simultaneously underline that little research has directly addressed food waste as a link between lifestyle and household size [23]. Small and single households tend to produce more food waste per capita than larger households [24,25,26], and these types of households are increasing in number, with young people making up a significant number of these households.

Quantities of food in too big packaging are a misfit for single-person households and young people’s lifestyles and are often outlined as a factor that leads to food waste. Evans [27] gave an ethnographic example of a person in their mid-20s named Tamsin and de-scribed her as “broadly typical of the single people, young couples and house-sharers” [27]. Living alone and having work that included traveling and erratic work schedules were aspects that affected Tamsin’s food provisioning. Tamsin’s food was wasted because it was difficult to find ingredients in smaller portions to cook healthy food from scratch, such that she ended up buying more than she needed. Furthermore, her food turned bad because she was often not home, as her work included a lot of travel and erratic scheduling. Moreover, this resulted in her not having the energy to cook food even when she was home. She then preferred to buy prepared food, while the other ingredients stored for a meal turned bad. The “proper” and “healthy” food made from scratch “imposes its own demands in terms of the timeframe within which it must be eaten”, which is understandably hard regarding adjusting to erratic work schedules when people are simply tired and hungry [27]. Another important issue experienced by this research participant was the lack of time and distribution for different social practices. Food stored at home also went bad and was wasted as Tamsin ate out with friends on the way home from work trips. This strategy was convenient for meeting them, as the train brought her to the city center where they worked. By going home to cook food, she would simply miss out on social life.

The case of Tamsin demonstrates two important aspects. First, eating healthy and cooking from scratch means buying many ingredients, but the packaging of ingredients in supermarkets is not suitable for a single-person household and leads to wasting food. More importantly, the case shows that food eating, keeping, and wasting practices also depend on broader socioeconomic contexts, such as work conditions. In her case, her erratic work schedule left little time and made it challenging to plan meals. The socio-temporal context of food practices is relevant for understanding the reasons for food waste.

This means that young people not only waste food because of their lack of knowledge or limited skills in cooking and the use of leftovers, but also because of time scarcity and their lifestyle, in other words, not only because of their individual choices, but also because of the broader structural issues and contexts in which they live. This was also observed by Revilla and Salet [28], who claimed that consumer practices in the kitchen alone could not be the only reason for their food waste. The overwhelming demands of everyday life are among the reasons for food waste, and having a focus on certain rituals at home and the social meaning of meals could also be factors that reduce food waste. Although this study did not specifically focus on young people’s lifestyles and households, it can be assumed that rituals at the dining table are transferred when young people move away from home.

## 4. Planning the Meal

Without underestimating the impact of social factors on lifestyle, Stancu et al. [29] identified food-related routines, such as planning, shopping, and the use of leftovers, as core drivers of food waste. These can all be related to purchase planning, where the theory of planned behavior (TPB) by Ajzen [30] describes an individual’s intention and ability to perform a certain behavior. The theory that has been widely applied to food waste studies [29,31,32,33] addresses three independent factors of intent.

The first is the attitude toward the behavior that is going to take place and refers to whether a person has a negative or positive attitude toward a specific behavior. In the case of young consumers’ attitudes toward the waste of resources and sustainability, there seems to be clarity in their minds, although the gap between attitude and behavior is clearly seen [34]. The author explained the gap as a lack of sufficient knowledge, and to obtain sufficient knowledge, people (young and old) must have a certain level of motivation, which was linked to involvement. The level of involvement of young consumers in food preparation and the purchase of these commodities seemed to be the core for understanding the attitude–behavior gap and should be linked to these young consumers’ awareness and trust in food providers.

The second factor in the TPB model is the social factor, which is called the subjective norm. This model refers to the perceived social pressure associated with performing a task in an accepted manner. Perceived social pressure is strongest if it is enhanced by a reference group, especially for socially constructed values such as sustainability and pro-environmental behavior [35]. Burlea-Schiopoiu et al. [36] found that families have a high degree of influence through moral and ethical issues, which are related to the norm of living life. The authors concluded that the more these young students are aware of this, the more they express it as a matter of their own morals. However, social norms are mediated through personal norms, and by that, any personal norm can become the main driver of sustainable behavior [34]. This means that even strong social pressure on a young person’s food purchase and common norms for avoiding waste will be overruled by the personal norm, which can be constructed by many other factors, such as finances, interest, ability to cook, or prioritized time for eating.

The third and last factor in the TPB model is the degree of perceived behavioral control, which refers to the subjective experience of accomplishing a behavior. This includes previous individual experiences and reflections on their advantages, disadvantages, anticipated obstacles, or inconveniences. It can be said that the easier it is to perform the task, the greater the intention of performing the task. Habits for frequent and repeated purchases might bypass the conscious level of intention and directly lead to action. In a Polish study by Przezborska-Skobiej and Wiza [37], it was found that students in the age group of 19-26 wasted food more frequently and in larger quantities than people aged 27+. One explanation for this was lower behavioral control, as these students had an emotional approach to shopping (cravings) and purchased food products without any pre-prepared shopping list (impulsive). Furthermore, the study showed that the group of students had greater food waste because they lacked ideas for using leftovers [37], indicating that leftovers do not become an issue when purchasing food for the next meal. This was also found in a study by Kymäläinen et al. [5], which showed that the challenges regarding food consumption habits in Generation Z were related to planning skills, impulse behavior, and lack of creativity with leftovers. In particular, leftover reuse routines were seen as the most important contributors to food waste. The biggest effect on food waste could be to change these consumers’ ability to change leftover reuse routines and the possible downstream effect on shopping routines.

The TPB model has been criticized for its simplified conceptualization, for missing the complexity of the human mind and behaviors [38], and because an intention does not necessarily lead to behavior [39]. Further, the model describes an action for achieving something or fulfilling a need, whereas an action for preventing something such as food waste might only have similarities to the described behavior. Being aware of the model’s limitations in food waste behavior, the three drivers of planned behavior can be used as a framework to assess food waste interventions and young people’s motivations and responses toward these interventions.

### Unforeseen Factors Affecting Food Waste

Sometimes, changes in behavior occur because of conditions that were not planned or that were completely external, such as the COVID-19 crisis. In a study by Burlea-Schiopoiu et al. [36], the authors showed how the pandemic crisis has changed young people’s perception of food waste and how it has led to increased ethical awareness of food waste. This study tested students’ perceptions through surveys and did not check for real changes in their behavior. The authors emphasized that the moment of a pandemic crisis might be a good moment to start changes. However, the study did not test how long the effect on food waste prevention from a crisis would last or whether the effect would be similar for segments other than students.

In a similar study by Jribi et al. [40], the authors observed that people in the COVID-19 lockdown rethought their food purchase behavior toward an increased focus on responsible consumption and, therefore, reduced food waste. It has also been found that “strong short-term fluctuations and changes” in lifestyle and eating habits could have a negative impact on food waste generation [41]. The scenarios in the study revealed that some households would produce more food waste due to a changed diet regime or a lack of foresight, while for others, it would be reduced due to the fear of recurrent purchases.

Finally, different types of crises have not been tested, and even though COVID-19 can be seen as a health crisis, it can also be experienced as a financial crisis, which calls for further research to clarify the impact on food waste from different perceptions of a crisis—small or big, personal, or societal. Whether a crisis can work as a catalyst for young consumers and enable them to start practicing responsible and sustainable behavior, mirroring what they express and how they want to live, is beyond the scope of this study.

**Figure 1:**
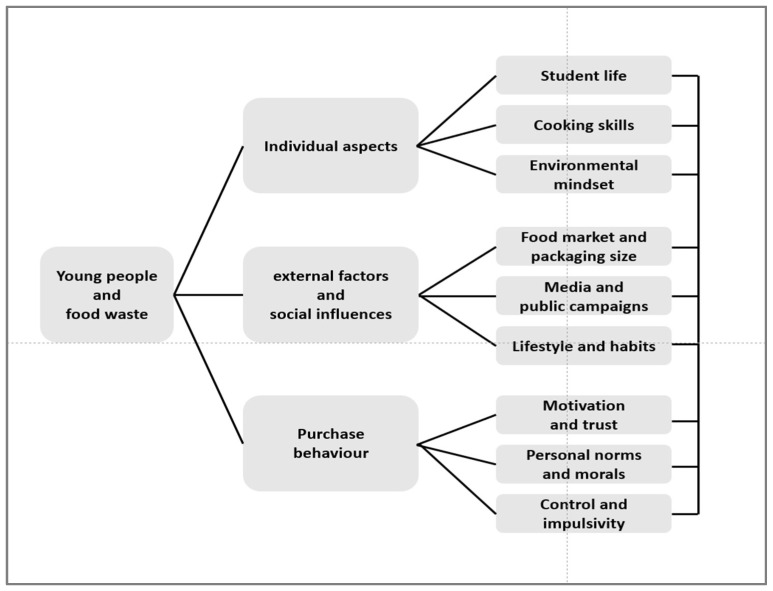
The conceptual model for young people’s food waste.

Summarizing the literature review on food waste among young consumers, Figure 1 gives a graphical overview, showing groups and sub-groups. It is important to regard the figure as an overview, which omits the nuances in causes of food waste among young consumers. Thus, the figure does not show the interrelationships that may exist between the causes, which is most often the case, since not one cause alone can explain the problem.

## 5. Interventions to Reduce Food Waste

According to Hebrok and Boks [42], there is a lack of diversity in studies on the effects of food waste interventions suggested in the literature. Interventions can be divided into: (1) technology that helps people plan, share, and maintain an overview of stock, (2) packaging and storing solutions that extend shelf life, and (3) information and awareness campaigns [42]. Reynolds et al. [43] divided intervention types into somewhat similar categories, although they emphasized the need for interventions based on changes within the political, systemic, and practice spheres.

Information-based interventions to change consumer food waste have been applied in several studies, including those by Whitehair et al. [44], Martins et al. [45], Stöckli et al. [46], Pinto et al. [47], and Soma et al. [48]. Campaigns based on the educational approach with the desire to improve knowledge on cooking skills have also been suggested, but there is generally a lack in evaluating these campaigns’ effectiveness among real consumption and over a longer period. In most research setups, it is difficult to evaluate the duration of an intervention through campaigns. Stöckli et al. [46] provided several examples of food waste intervention practices that lack systematic evaluation, which in many cases, are caused by the methods used.

Furthermore, most of the reviews regarding food waste have focused on the reasons for and causes of food waste rather than interventions to solve the food waste problem [42]. The authors outline in their literature review of 112 scientific publications about food waste design interventions in households that there is little diversity in food waste interventions. Many intervention designs are based on prototypes, some as suggestions for improvement and others on pure speculation.

The authors found that most of such interventions for solving food waste were related to packaging design and storage in refrigerators or freezers. Interventions based on packaging are seen in different forms, going from studies with the packaging being able to preserve food better to studies where information about food waste prevention is given on the packaging. Date labelling is an example and has long been known as one of the culprits behind food waste [23]. New types of data labeling, even with digital technology and color coding, have been introduced as a way to inform and learn new consumer habits [42], although it remains to be proven whether these interventions will influence food waste. Technology-based interventions, such as fridge cameras or apps, have been reported to be effective in reducing food waste, but without accurate quantification [42].

The type of intervention made to increase awareness through information falls within the campaign category. Information alone seldom changes consumer behavior and tackles the food waste problem [46], which was also described in the review by Principato et al. [22]. The authors found that the most successful intervention campaigns are those targeting subjective norms, and they emphasized that food waste campaigns based on subjective norms should be able to utilize the tendencies of individuals and conform to peers’ actions or beliefs. In particular, campaigns using comparisons with other people in the same situation as the target group seem to reduce food waste. Informational interventions are often used but have a relatively low effect, and they emphasize the need for a combination of intervention types, which is most often the conclusion ending review papers on food waste.

### 5.1. Systemic Interventions

According to Principato et al. [22], consumers, companies, and institutions should cooperate to minimize food waste, which was also the argument by Baron et al. [49], who argued for a service ecosystem that is able to grasp situations where food waste becomes prominent. Viewing food waste from a societal service innovation perspective, the authors argue that the role of institutions such as retailers, producers, and authorities and their interrelations become a way to challenge the existing system with its obvious food waste issues. Packaging overlaps several links in this product–service system and plays an essential role in people’s in-store decision making [50]. Several studies have focused on how packaging can have a positive impact on reducing consumer food waste, either by prolonging shelf life, protecting food, creating awareness, or affecting home use. Based on the study by Williams et al. [26], 20–25% of wasted food in households in Sweden could be caused due to packaging functions that do not meet consumer needs, such as being too large or being difficult to empty.

Technological solutions should be mentioned in the holistic view of food waste. Various kitchen gadgets have been suggested to ease the measurement of portions (e.g., pasta and spaghetti) to prepare the optimal portion of food and to reduce possible leftovers. Other digital solutions in the form of mobile device applications have been invented to increase the visibility of food waste behavior and reflections [51], using photos of food leftovers that would go to the bin. A good example of such a solution is BinCam, which was created as a social persuasive system with the intention of motivating reflection about food waste generated at the household in a playful manner and to change food waste generation and recycling habits of young adults [52]. Generation Z is often described as digital natives, so it is expected that various applications could be an obvious tool for intervention in food waste reduction among young people.

### 5.2. Intervention Targeting a Young Segment

Very few studies on food waste interventions have specifically targeted young consumers. Based on the study by Kymäläinen et al. [5], there are three ways to enhance sustainable food consumption behavior among young consumers: (1) support a shift in diet, (2) provide concrete information, tutoring, and education, and (3) encourage sustainable food consumption. This is in line with findings from Burlea-Schiopoiu et al. [36], showing that the more knowledge young students have, the higher their intention to reduce food waste. Knowledge does not need to be in the form of campaigns, as sources of knowledge are diversified and could include observed behaviors, trial and error, or peer-to-peer conversations [36].

Few studies have been conducted on food waste and nudging in the household environments [53]. According to the authors, young people and those living in large households with young people are open to changing their behavior with nudging methods. However, the respondents were mostly interested in feedback regarding their own individual behavior (quantities and financial costs), social exchange and interaction, and specific advice on meal planning.

### 5.3. Effectiveness of Interventions

Reynolds et al. [43] found 17 effective interventions for food waste reduction within households and institutions, with professional staff in charge of preparing and serving food. Plate size reduction at hospitals was among the most effective, followed by changes in the nutritional guidelines at schools. This is in line with findings from Kallbekken and Saelen [54], showing that reducing plate size could reduce approximately 20% of food waste in hotel restaurants in Norway, which again was outlined by Freedman and Brochado [55], showing that reducing the portion size is a way to reduce plate waste in restaurants.

The United States of America National Strategy to reduce food waste at the consumer level [56] highlighted seven intervention types that can contribute to reducing consumer food waste: (1) appealing to values, (2) engaging consumers, (3) evoking social comparison, (4) providing feedback, (5) providing financial incentives, (6) modifying the choice architecture (i.e., nudges), and (7) providing how-to information.

From these choices, architecture and social comparisons were found to be the most effective ways to influence consumer behavior. Intervention choices were based on studies that have examined consumer behavior in the following domains: energy conservation, water conservation, waste prevention/management, recycling, diet change, and weight management.

### 5.4. Limitations with Interventions

Many authors have outlined the basic limitations of intervention studies on food waste. In a study of the impact of a crisis on food waste perception [36], the authors described the effect over time as a limitation. The effect of COVID-19 on food waste could have a here-and-now effect, but it could evaporate when things become normalized. This was also the conclusion in a review by Reynolds et al. [43], who found that campaigns, cooking classes, food sharing apps, and sharing through apps influenced food waste reduction, but with very little or no robustness. The lack of reproducible and quantified interventions is a core challenge in many food waste interventions, and intervention studies with better and standardized guidelines are needed [44]. The authors describe the difficulties in making evidence-based decisions to prevent food waste as a significant evidence gap, and they see this as a barrier to change behavior.

The most common and pervasive limitation is related to the impact of the intervention on the actual amount of food wasted, as it is difficult to measure the actual amount of food wasted before and after the intervention. A method capable of measuring the exact amount of waste by weighing has been used in the garbage stream of households, cantinas, etc., but it is difficult to apply it to food waste, as it does not measure what could be poured down the drain, fed to pets, or composted [57].

Several papers cited in this study have emphasized some methodological and/or conceptual issues by not clearly distinguishing between household recycling and food waste [3]. The European Union Horizon 2020 project REFRESH [58] suggests a method for evaluating the effectiveness of interventions designed to prevent household food waste. The suggested method is based on understanding the intervention, development of the evaluation approach, dissemination, and implementation of the findings. The project report concluded that there is a need for thorough and logical mapping to outline the design of activities in the intervention and to register the final outcome. The first step was to define the aim of the intervention by answering the following questions: What does the intervention seek to obtain? Who does the intervention seek to influence (the audience)? What type of intervention is used (single/multiple or short/long-term interventions)?

**Figure 2:**
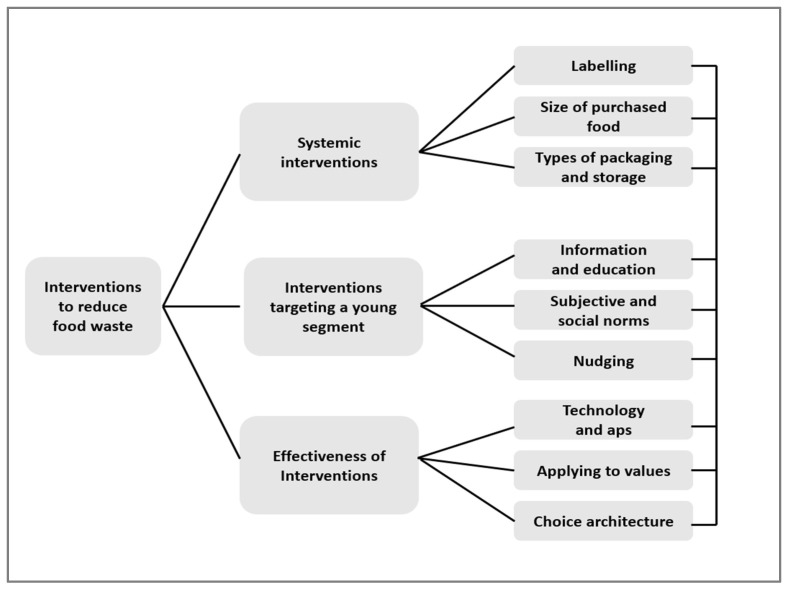
The conceptual model for interventions against food waste.

Figure 2 outlines the key interventions used to reduce food waste. These interventions should be combined, keeping in mind the delicate interlinkages between them and the possible synergies when several interventions are applied simultaneously.

## 6. Relating Literature Review with Youths’ Perception of Their Own Situation

Young consumers accept the issue of food waste and have several experiences in their own lives. Even though they see themselves as responsible for the environment and for climate change, they also acknowledge that there is a challenge with food waste in their own household. To strengthen the understanding of the way young consumers interact with food and to discover strategies to decrease avoidable food waste in three Nordic–Baltic countries (Lithuania, Finland, and Denmark), the project “Let’s not waste food” was implemented.

To design an effective intervention, it is important to seek knowledge about similar interventions in similar contexts. As intervention usually requires flexibility since it is usually not possible to implement an intervention plan exactly as planned; the setup and the plan for evaluation need flexibility. For the later dissemination of findings, it is relevant to define types and amounts of resources, and finally, it is relevant for future interventions to register what was carried out, when and who was involved, and conflicts of interest, which must be built on insights from the actors themselves. With this starting point, new insights from young people’s own ways of living require an exploratory scientific approach, which we find in inductive research.

Based on the literature review on food waste among young people (Figure 1), and potential interventions to reduce food waste (Figure 2), a protocol for focus group interviews was developed, and the setup in each country followed the same protocol with the aim of obtaining more insights from young Nordic–Baltic people’s expression of food waste and their own thoughts on how to deal with the challenges.

Nine focus groups were organized in Lithuania, Finland, and Denmark, that in total summed up insights from 50 young people. The focus groups were composed so that the participants represented young people in their own households. The participants should be responsible for the main food purchase, and they should live alone or with roommates/partners, but there should be no children in the household. The participants should be financially independent, meaning that their main income should come from their own work. There were no requirements for education level, nor for food preferences or diets. The focus group setup strived to have gender equality and an age span between 18 and 26 years.

People were invited by mail to an optimal focus group size of around five to six people per session. In the mail, people were also informed about the topic for the interview, and our code of ethics provided us with guidelines to inform participants to participate only according to their free will. They were further informed that the interview would be recorded and that all data would be anonymized. Lastly, they were informed that the use of data was exclusively for scientific publications. The recorded data were stored on servers protected by passwords and were only available for members related to the project.

### 6.1. The Data Outlined Five Scenarios

The recorded interviews were transcribed, and the data were analyzed in the first round on three levels: (1) what the respondent said, (2) what the respondent meant, and (3) the consequences of the statement [59]. This method was applied to all transcribed interviews and then joined into one file. The first data analysis yielded several scenarios, which were then condensed into four scenarios:

- Special occasions;
- Assessing food quality;
- Kitchen habits;
- Shopping habits.

Before outlining these four scenarios further, it is relevant to define the terms we used. We have theoretically referred to the issue of cooking skills, which means that a person can prepare food for consumption. This could be professional cooking skills or domestic cooking skills, and while analyzing our focus group data, we broadened the interpretation of domestic cooking skills and constructed categories from the data, and not the other way around.

Analyzing data through these levels of domestic cooking skills enabled us to see the informants’ sensory and perceptive ability to assess food quality and its edibility, as many relied more on the label information rather than on their own senses. Kitchen habits such as preparation, serving, and storage are important when avoiding food waste. Further, planning the meals, purchasing situations, and even the ability to use leftovers are related to cooking skills. This emphasizes the value of seeing the cooking process as a whole, from food acquisition, through cooking, to leftovers utilization.

### 6.2. Food Waste Related to Special Occasions

Young people often seem to be in a situation in which plans are being changed. This change in plans causes a mismatch between planned food purchases and eaten food, which results in food waste. The respondents acknowledged the problem but at the same time had difficulty seeing the solution to unforeseen events. A respondent from Lithuania expressed the concern this way: “… *we try to do something about it, but sometimes you just can’t*”.

The reasons for not being able to control these unexpected changes in plans are based on circumstances that are beyond the person’s influence. One example of an occasion could be an invitation from the family, a situation at their job, or friends calling for an unplanned trip into the city. A respondent from Denmark expressed the situation by: “… *then you might not be right at home to eat the leftovers …*”, which can be supplemented by a Finish statement outlining work-related reasons for food waste, “*… then we buy food for a longer period, and then sometimes I have to leave suddenly from home because of my work*”. These findings are in line with the research by Evans [27], where life as a mid-20 year old, typically a single person with an erratic work schedule that includes traveling, might be a reason for wasting food. From Lithuania, it was described as an unexpected business trip, which was followed by, “*… then coming back in a week … you have to throw it away*”.

Eating out at a restaurant is a special occasion where participants often experience food waste, because there is more food than they can eat. The informants seemed to have two different ways to solve this issue. Either they eat everything, or if this is not possible then, “*… I ask for some sort of boxes there and everything is fine*”. Taking leftovers from a restaurant and bringing them home is not common in Scandinavia, and this statement from Lithuania might also express a wish for a cultural change in the view on leftovers when people are eating out.

Not surprisingly, the informants in this study came up with *better planning* as the first obvious solution to this unforeseen situation. However, the respondents also acknowledge that this might not be a long-lasting solution. A Danish respondent put it this way, “*I think it requires a structured everyday life … I do not have it with my studies and new work*”. Another potential solution can be found in the statement, suggesting that several people prepare food together and eat together, which taps into what is seen as the level of cooking skills. When an unforeseen situation occurs, then there will still be others in the food network to prepare the purchased food and have it for dinner. This is expressed in a statement such as, “*…least it made me join several food clubs… such social things around and eating together…*” This last part of the comment points to another issue in young people’s lives, which is the social dimension.

Unexpected situations are also facilitated by the young people themselves. Spontaneous events happen in young people’s lives, and for them, it is important to be part of the social network. Knowing that there is food at home, an ambivalent experience easily arises, where on the one hand, food should not be wasted, but on the other hand, friendships must be kept active. In this situation, food waste seems to become second priority, as expressed in a statement where the person expresses being somewhat ambivalent about the fact that she wants to save money and not throw food out, but she really does not want to say no to social networking.

Having the best intentions not to waste food in these unexpected situations does not seem to be enough to avoid food waste. A respondent from Finland expressed that even though she has planned the food and stored it in the fridge, because of an unexpected situation that changed her plan, it became food waste anyway, “*… especially some vegetables easily get old*”. Even leftovers can be wasted due to unexpected things to happen, as the plan of eating leftovers might be challenged by unexpected things to happen. “*Then those leftovers might also sometimes go to the trash…*” says a respondent from Denmark.

Zhang et al. (2021) [4] also outlined the issue of utilizing leftovers as a reason for food waste in Generation Z. In that paper, food waste was related to young people’s low tendency to cook for themselves and by that to utilize leftovers, but findings from these focus groups placed more nuance on the reason for food waste. Food waste seems to stem from a combined level of kitchen abilities and unforeseen events in young people’s lives.

### 6.3. Food Waste Related to Assessing Food Quality

The informants found it difficult to assess the prepared food. When the meal is stored in the fridge, it becomes a challenge to evaluate whether the food is edible or not. Uncertainty in their judgement causes a feeling of potential food waste. This is because it is difficult for the informants to judge whether the food was inedible and should be thrown out or whether it was fine. This uncertainty results in a better-safe-than-sorry argument, and the food will be thrown out to stay healthy.

Whether to discard a food product depends on more than health facts, as the price paid for it influences the evaluation in such a situation. When clear mold stains are visible, this is a clear sign of inedible food; however, the price still influences the decision to it throw away. A Danish informant expressed it this way: “*If there is mold at one end and the other end is fine… I would probably do that (*throwing out*) with a carrot or something like that… not with sweet potatoes because I think they are too expensive*”. The informants fully acknowledged the bad conscience that comes with judging food and making decisions based on emotional and irrational judgments, which is expressed in the statement: “… *every time you throw food out, you feel like a Satan*”. To come across such unpleasant feelings, one solution, which is both acknowledged as irrational and a kind of self-irony, is to let the food stand for a couple of weeks in the fridge, because clearly rotten food simply has to be thrown out: *“… sometimes I let it stand for a week because I do not like to throw it out… then it get even more rotten before I throw it out”*. This underlines how uncertainty and emotions about eating “old food” are reasons for food waste.

Adding labels to the packaging does not seem to be a solution with uncertainty in evaluating food. The informants expressed difficulties in decoding the labels on the packaging. Even though labels such as Swan label have been on packaging for a long time, informants expressed uncertainty in knowing the full meaning of the labels and what they stand for. This uncertainty in decoding packaging information can be supplemented by other value labels, such as organic or animal welfare. The informants know about the many and sometimes too many labels, which result in another kind of uncertainty. The informants had difficulties in seeing interlinks between different labels and could not weigh animal welfare against food waste. Knowledge of labels might provide important guidelines, but at the same time, too many labels and guidelines will open an uncertainty in the hierarchy on which one is the most relevant, and guiding labels can be a double-edged sword.

Relating this to the findings in the review by Principato et al. [22], who describes the most successful intervention as being those targeting the subjective norms, points to the limitation in humans’ capacity to deal with several serious issues simultaneously. This might then result in a more irrational conclusion, where one inappropriate action can be offset by another good action. Among informants, better cooking skills are suggested for solving issues when subjective feelings and experiences cause food waste. In particular, a better selection procedure during a purchase is stated in response to keeping subjective and idiosyncratic assessments out. Informants with good cooking skills do not look up the expiring data on the packaging, and it can even be utilized to avoid food waste in retail. “… *I sometimes even look in the shops for some bread already there* (at the expiring data) *… I’m just sorry to see it being thrown out*”. Another informant from Lithuania shows how experiences from preparing food help to reduce food waste: “… *I always taste and smell*”, and this is emphasized by the statement on explicit knowledge about specific ingredients used for cooking, “*… products that are high in sugar, they have natural preservatives …*”.

However, good sensing and cooking are not the only habits that can prevent food waste. Consumers have many different kinds of goals related to food (in addition to food waste), such as eating safe food and enjoying food [27,42], time spent on making purchases, and preparing food, and the personal benefits associated with food (i.e., hedonistic goals) are more important than normative ones (such as reducing food waste) [60]. People desire to eat different types of food, and from Finland, it was expressed: “… *I want to eat different kinds of food and then sometimes it is challenging to eat all that food* (before it becomes spoiled) …”. A similar situation was pointed out by the Lithuanian informant: *“… it’s very hard for us to plan to consume those leftovers, because those desires usually change every day—one day you want something with a lot of vegetables, the next day you want with a lot of meat”*. Moreover, the informants do not like warmed up food, which makes the consumption of leftovers more difficult. However, an interesting observation is that, for these reasons, informants try to buy only what is needed for one meal and follow the portions of food in recipes: *“…we usually look at recipes to make something new, we see that according to the recipes to buy as much as you need to use everything at once…”*.

### 6.4. Food Waste Related to Kitchen Habits

The main reason for food waste provided by informants was poor planning. It was expressed in the idea that better meal planning can decrease the amount of food waste expressed by an informant from Finland: *“… so I have not planned up a schedule for my eating and dining … anyhow I have reserved something to eat from my fridge … and then my mind can change, and I don´t want to eat what was in the fridge … no planning and hurry, I would say that is the key reason“*. A study among young students in Poland showed that young men are more optimistic than women regarding their own household ability to generate less food waste when planning grocery shopping and eating all purchased foodstuffs [61]. The authors raise the explanation that such phenomena could be explained by men having less responsibility and experience in shopping, cooking, and cleaning up for leftovers. This refers to young people’s many different activities and responsibilities in their lives; for some segments, food purchase, meals preparation, and planning may not be a top priority. This could be the way of describing time scarcity and is nicely illustrated by a Finnish informant: *“… when I have time, I don´t use it to think what kind of food I will buy … rather I use the time for something else”*.

The incorrect calculation of the amount of food in the packaging is a matter of planning and is mentioned as a reason for wasting food. Big packaging are interpreted as offering a lower price per kilogram or more liters of the product, although the informants are not able to consume it. “*I will buy the package of 2 L since it is cheap, but then I will not use it*” says an informant from Finland, and in Denmark it is illustrated by, *“… good offers on those big packages, so sometimes you buy them, but then you do not get to use it all”*. The wrong calculation of how many portions will be consumed from prepared food is mentioned as an example of food-inappropriate planning. Typically, vegetables, dairy, and meat products are wasted because of their large packaging or portion size. This was also found by Williams et al. [26], showing that 20–25% of household food waste is caused by excessively large packaging or packaging that is difficult to completely empty.

The ability to eat all purchased food during a single serving is not always possible. This was expressed by an informant from Finland: *“… those products that is used just a little amount, but you can´t buy them in little packages, like curry paste or pesto … then you use them just once and the rest is not used”*. The informants asked for information or suggestions on how to organize the process of purchase and meals during the week to avoid food wastage.

### 6.5. Food Waste Related to Shopping Habits

Shopping and meal planning have been identified as ways to reduce food waste [31]. Many Finnish informants pointed out that going to a store without a list is one of the causes of food waste. *“At the grocery store I start to wonder which food products we have at home and then at home. I then notice that we have already 10 packages of those products”*. Making a shopping list, on the one hand, helps for faster shopping, but at the same time, it is mentioned as a way to buy food that will not be fully used: *“… I never make a shopping list, because it seems that if I did it, I would buy what I don´t necessarily need, or which I might not even use”*, expressed one of the Lithuanian informants. Shopping when hungry, shopping spontaneously, or being exposed to a good offer can cause food waste *“… sometimes you want something, you see something delicious, you just buy it and so on”*, said a Lithuanian informant, and followed up by, *“… sometimes you buy them, but then you do not get to use it all… then it is really such a waste”*.

The informants expressed the need for skills to plan their purchases. The new technological solutions that can help to measure a suitable amount of food is suggested by an informant from Finland: “*application which could help to evaluate how much food is needed for different meals*”.

### 6.6. Supplementing Findings

New ways of living change the perception of how much food is needed, as well as being able to use leftovers, planning. etc. This process could be affected by other’s (surrounding persons) attitudes to food waste, explained by an informant from Finland, *“… my girlfriend, she noticed that I was throwing a lot more food than she in the trash. My girlfriend suggested that every time I throw food in the garbage, I put money in a bowl and see how much has accumulated. The bowl is full now”*.

Joint actions such as food sharing could be relevant for young people who have started living alone, which is expressed by the Lithuanian informant: *“the problem is that when I make only for myself some of the food and products are left, it seems to me a good solution is to share it with others … because one doesn’t use everything”*. Social media can work as a way for people to find others with whom to share food or meals. Thus, it is good to have places such as a joint kitchen in a dormitory or a social place in the city where everyone can share surplus food or meals without any limitations. A national or worldwide agreement to decrease food waste has also been mentioned as a motivational factor. In particular, big players, such as supermarkets or restaurants, make an effort to reduce food waste.

**Figure 3:**
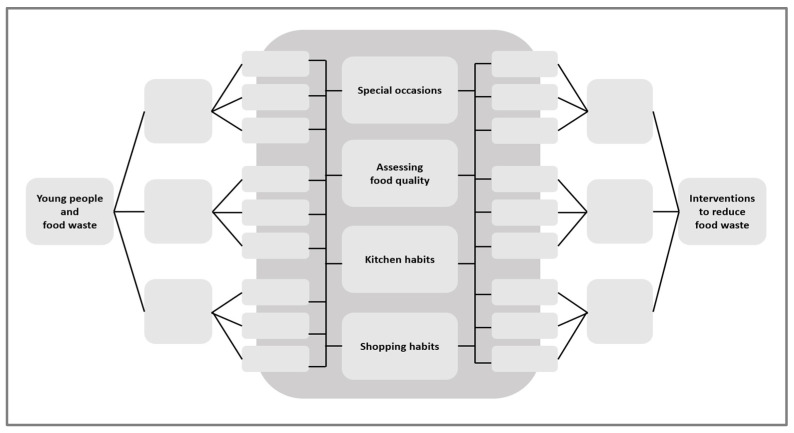
The relation between the reason for food waste among young consumers, interventions, and young people’s own reflections on potential solutions.

Placing reasons for food waste among young consumers, potential interventions to reduce food waste, and findings from young people’s own view on food waste and potential solutions, Figure 3 gives a graphic view of the relations. The figure, which includes Figure 1 and Figure 2 (mirrored), should be read as an overview of relationships lacking nuance.

## 7. Potential Interventions and Need for Further Research

Food waste occurs within many different but interconnected practices of everyday life, such as shopping routines, storage, cooking, and eating. People are not aware of all the drivers behind the food they waste because they are deeply entangled in everyday routines. Finding the best ways to influence, change, and limit food waste through interventions is not a straightforward task. Findings from previous studies have shown a number of possibilities as well as many challenges and limitations. Finding the best intervention should always be based on the documented best practices, although this is also a weak aspect of most food waste studies. This paper has outlined what has been documented in academic papers and may oversee good practices that have not been published or documented in the journals selected for this study. The empirical data from nine focus groups outline young people’s views on their own food waste, and at the same time, they give insights into young people’s suggestions for possible solutions. The nine focus groups were in three Nordic–Baltic countries, which provided a variety but at the same time limited option for generalizability. Despite these limitations, the following interventions can be considered as recommendations for potential future research among young consumers.

### 7.1. Intervention Related to Special Occasions

Knowing when food waste appears and focusing on interventions at a specific time in young people’s lifestyles, based on the young people’s words and findings from the case study by Evans [27], show that young people’s food waste could be linked to their work situation and lifestyle. A potential intervention would benefit from taking a starting point in the study by Aschemann-Witzel et al. [11], in which lifestyle patterns were linked to food consumption and food waste. The importance of daily routines has been identified in food-related daily behaviors, and practices that cause food waste are strongly intertwined with daily routines [21,42].

Some informants during the focus group discussions suggested that eating together or sharing food with others may reduce food waste in unforeseen situations. Food sharing spaces, live or virtual, can be a solution to create wide networks of people with similar attitudes to food. Many sectors can play an important role in facilitating such spaces and practices. A food-sharing network could involve as many participants as possible to ensure the possibility that someone from the network will use the food surplus suggested by another participant.

Interventions within this field should, in small steps, change young people’s routines and outline their impact on food waste. The time when young people leave home and start a life for themselves could be an opportunity to test simple interventions. Changes to smaller plate sizes were found to be effective in reducing food waste in institutions but could be relevant to testing in small households. A case study based on a deductive research design could compare two identical groups (target vs. non-target) with different plate sizes (or similar kitchenware) and provide quantitative data on the effect on food waste. Planning an intervention at the time when young people establish their own households could be an advantage to research on this crisis [40], where the uncertainty of changes in society makes people change their view of food. The similarity between a crisis and the situation of establishing one’s own household should be addressed in a research design and is worthy of investigation. Young consumers could have brainstorming sessions in focus groups about the tools to reduce food waste by nudging at home (by utilizing, for example, technology opportunities).

### 7.2. Interventions Related to Assessing Food Quality

The attitude–behavior gap seems to be an obvious target for intervention. Young people already have a positive attitude toward the environment and do not need to be convinced of the food waste issue. Young people in our study accepted the gap between their wishes and their actions, and research has shown a link between attitude and the level of motivation [34]. For a young segment, it seems beneficial to link motivation to avoid food waste with food providers’ awareness and trust. A potential intervention study should build on findings from an international project, showing that young people trust specific local food providers [62] and investigate ways to increase trust and how it has impact on the amount of wasted food.

### 7.3. Interventions Related to Kitchen Habits

Young people still need more knowledge and skills, such as how to store fresh vegetables and fruits for a longer period by using proper temperature, humidity, or suitable packaging. Some practices were already known for them such as food freezing, but skills on how to use frozen food later were missing, for example, one Lithuanian person said, “*freezer is food cemetery, food is frozen and later thrown out*”.

Whether abilities in the kitchen lead to food waste or not, these abilities seem to originate from home. Informants from Lithuania are very clear about this and express how shopping and preparing food together with their parents have made the fight against food waste possible because of the skills and knowledge they have gathered. Further research based on kitchen habits learned at home could be relevant for food waste interventions targeting the young population, and there are calls for a longitudinal research design.

### 7.4. Interventions Related to Shopping Habits

Interventions that target food purchase planning should focus on the topic of being in control. This study found that a lack of control over the time of food shopping can be linked to food waste [37]. This lower behavioral control can be related to shopping habits, which can be further described as impulsive purchases. An intervention aimed at increasing behavioral control and avoiding impulsive purchases could investigate the effects of preparing and using a shopping list. The test could investigate the physical list against a digital list with extended features, such as food stored at home or even leftovers, which was seen as an important contributor to food waste. Research on using a shopping list can be an intervention in a basic case study, in which two identical groups (target vs. non-target) can be compared and easily provide feedback.

Campaigns and information interventions on food waste using social norms seem to have an effect. Social pressure seems to be strongest if it comes from a reference group, and especially from a group to which the perceiver of the information is related [36]. Campaigns that utilize a high degree of moral and ethical pressure enabling the young segment to express this as a matter of their own morals should be tested. In such a research design, which could be both inductive and deductive, it is relevant to investigate and include personal norms, as this will overrule the social norms, and a campaign based on social norms could be worthless or could even backfire. Although the research outlines limited results from campaigns, it should not be neglected as a potential intervention.

The retail sector plays an important role in the interface between the consumer and the production chain [11]. In the focus group, participants brainstormed on how the retail sector could support young consumers in reducing food waste. In addition, the possibilities for online shopping could be discussed. An intervention study could take the point of departure in the USA National Strategy to Reduce Food Waste [56] and look into the effect on “how to” as a means for changing behavior through so-called easy, everyday tips. In this type of intervention, it would be relevant to utilize young people’s willingness to use mobile applications, either as a nudging feature or documenting feature.

## 8. Contributions and Discussions

Research has shown that combined interventions work best and that a discursive tension between food waste, food safety, and a healthy diet should be further investigated. Any kind of research on food waste must take a holistic view of the problem and see it in the context of other challenges in society. In this study, we have focused on young people showing the complexity in the problem and outline that one intervention cannot stand alone. There are similar challenges in other segments, where one cause cannot explain the entire challenge, and where one solution or intervention does not work in isolation.

Hence, it could be proposed that one intervention could be a campaign on how to combine sustainability of food with healthy and safe nutrition, in order to become wiser on this tension. However, it is stated that the effectiveness of information campaigns is uncertain, and this intervention could take a more direct form of workshops and seminars where young people can express and demonstrate their practical approach to this tension between common environmental issues and personal health issues.

Many interventions take a top–down approach in order to control, nudge, or increase the awareness of food waste practices through laws and regulations. An important approach to the phenomenon of food waste requires an explorative methodology. The bottom–up approach, in which the target group puts their own words on the phenomenon and even gives their view on one or several solutions, was taken in this study and could be mentioned as a way to change behavior. Just taking part in the debate might cause small changes in people’s mindsets, and behaviors might change as a consequence of challenging one’s own words. This type of intervention take time but can easily be tested.

Generally, people agree that packaging preserves and protects food, and its impact on food waste can be explained and understood. However, in people’s minds, it is unclear what type of packaging is best for the environment. Reducing packaging material or making it biodegradable is at first glance positive, although less packaging may increase food losses. When it comes to a more complicated trade-off between the environmental impacts of packaging and food losses, it becomes impossible for ordinary people to perform this type of calculation. What goes on in the consumer’s mind when a decision is made in-store might be very different from a complicated CO_2_ calculation. In the case of cheese with high CO_2_ impact, an increase in packaging can be justified in order to preserve it. On the other hand, increased use of packaging material for preserving ketchup with a relatively low CO_2_ footprint cannot be justified. There is a need to understand in-store decisions and how purchase habits can be influenced in a way that they will reduce food waste. This involves several stakeholders in the supply chain, which might challenge the simplicity of such research and blur the outcome.

In the EU Horizon 2020 project REFRESH [58], a protocol and method with clear evaluation criteria was described as the need for better research and interventions to prevent household food waste. It is important to clarify the expected outcomes and to understand the limitations of the interventions. Regardless of the type of intervention, a logical mapping that outlines the research design, activities in the intervention, and registration of the outcome is paramount.

Interventions usually require flexibility, as things may not go as planned. Unexpected effects on food waste are reported in this study as the core reason for food to be wasted. The COVID-19 crisis had an unexpected effect on people’s perceptions of food and food waste. There may be other unexpected features or other situations that have not yet been documented. For later dissemination, it is relevant to specify the circumstances and the time for the effect of an intervention to last. Although the field of research within food waste is not new, the number of published successful interventions is limited. Therefore, it is advisable to initiate simple and small-scale interventions. Interventions on food waste deal with lifestyles, and few people are willing to accept radical changes in their way of living. The interventions outlined in this paper and young people’s own reflections on what might work for them can, as a starting point in further research, be ranked in relation to methodological simplicity and ease of implementation.

## Data Availability

The data used to support the findings of this study can be made available by the corresponding author upon request.

## References

[1] Su C.-H., Tsai C.-H., Chen M.-H., Lv W.Q. (2019). U.S. Sustainable Food Market Generation Z Consumer Segments. Sustainability.

[2] Goöçer A., Oflaç B.S. (2017). Understanding young consumers’ tendencies regarding eco-labelled products. Asia Pac. J. Mark. Logist..

[3] Bravi L., Francioni B., Murmura F., Savelli E. (2020). Factors affecting household food waste among young consumers and actions to prevent it. A comparison among UK, Spain and Italy. Resour. Conserv. Recycl..

[4] Zhang J., Ye H., Bhatt S., Jeong H., Deutsch J., Ayaz H., Suri R. (2021). Addressing food waste: How to position upcycled foods to different generations. J. Consum. Behav..

[5] Kymäläinen T., Seisto A., Malila R. (2021). Generation Z Food Waste, Diet and Consumption Habits: A Finnish Social Design Study with Future Consumers. Sustainability.

[6] Madkulturen, Adolescents’ Shopping and Eating Habits—A Study of What Creates Food Waste among Young People and Possible Keys to Avoiding It (in Danish). https://www.madkulturen.dk/wp-content/uploads/2021/05/Unges-indkoebs-og-madvaner-Mindre-madspild-blandt-unge-Maj-2021.pdf.

[7] Porpino G., Parente J., Wansink B. (2015). Food waste paradox: Antecedents of food disposal in low income households. Int. J. Consum. Stud..

[8] FAO (2011). Global Food Losses and Food Waste.

[9] Roodhuyzen D.M., Luning P.A., Fogliano V., Steenbekkers L.P.A. (2017). Putting together the puzzle of consumer food waste: Towards an integral perspective. Trends Food Sci. Technol..

[10] Knezevic B., Kurnoga N., Anic I.-D. (2019). Typology of university students regarding attitudes towards food waste. Br. Food J..

[11] Aschemann-Witzel J., de Hooge I.E., Almli V.L. (2021). My style, my food, my waste! Consumer food waste-related lifestyle segments. J. Retail. Consum. Serv..

[12] Nikolaus C.J., Nickols-Richardson S.M., Ellison B. (2018). Wasted food: A qualitative study of US young adults’ perceptions, beliefs and behaviors. Appetite.

[13] Olavarria-Key N., Ding A., Legendre T.S., Min J. (2021). Communication of food waste messages: The effects of communication modality, presentation order, and mindfulness on food waste reduction intention. Int. J. Hosp. Manag..

[14] Brown K.W., Ryan R.M. (2003). The benefits of being present: Mindfulness and its role in psychological well-being. J. Personal. Soc. Psychol..

[15] Holtzman J.D. (2006). Food and Memory. Annu. Rev. Anthropol..

[16] Murcott A. (1999). Scarcity in Abundance: Food and Non-Food. Soc. Res..

[17] Mintz S.W. (1985). Sweetness and Power. The Place of Sugar in Modern History.

[18] Blichfeldt B.S., Mikkelsen M., Gram M. (2015). When it Stops Being Food. The Edibility, Ideology, Procrastination, Objectification and Internalization of Household Food Waste. Food. Cult. Soc..

[19] Goody J., Counihan C., van Esterik P. (2013). Industrial Food: Towards the Development of a World Cuisine. Food and Culture. A Reader.

[20] Watson M., Meah A. (2012). Food, Waste and Safety: Negotiating Conflicting Social Anxieties into the Practices of Domestic Provisioning. Sociol. Rev..

[21] Evans M.D. (2014). Food Waste. Home Consumption, Material Culture and Everyday Life.

[22] Principato L., Mattia G., Di Leo A., Pratesi C.A. (2021). The household wasteful behaviour framework: A systematic review of consumer food waste. Ind. Mark. Manag..

[23] Halloran A., Clement J., Kornum N., Bucatariu C., Magid J. (2014). Addressing food waste reduction in Denmark. Food Policy.

[24] Silvennoinen K., Katajajuuri J.-M., Hartikainen H. (2014). Food waste volume and composition in Finnish households. Br. Food J..

[25] Jörissen J., Priefer C., Bröutigam K.-R. (2015). Food Waste Generation at Household Level: Results of a Survey among Employees of Two European Research Centers in Italy and Germany. Sustainability.

[26] Williams H., Lindström A., Trischler J., Wikström F., Rowe Z. (2020). Avoiding food becoming waste in households—The role of packaging in consumers’practices across different food categories. J. Clean. Prod..

[27] Evans M.D. (2012). Beyond the Throwaway Society: Ordinary Domestic Practice and a Sociological Approach to Household Food Waste. Sociology.

[28] Revilla B.P., Salet W. (2018). The social meaning and function of household food rituals in preventing food waste. J. Clean. Prod..

[29] Stancu V., Haugaard P., Läheenmäki L. (2016). Determinants of consumer food waste behaviour: Two routes to food waste. Appetite.

[30] Ajzen I. (1991). The theory of planned behavior. Organ. Behav. Hum. Decis. Process..

[31] Stefan V., van Herpen E., Tudoran A.A., Lähteenmäki L. (2013). Avoiding food waste by Romania consumers: The importance of planning and shopping routines. Food Qual. Prefer..

[32] Graham-Rowe E., Jessop D.C., Sparks P. (2015). Predicting household food waste reduction using an extended theory of planned behaviour. Resour. Conserv. Recycl..

[33] Visschers V.H.M., Wickli N., Siegrist M. (2016). Sorting out food waste behaviour: A Survey on the motivators and barriers of self-reported amounts of food waste in households. J. Environ. Psychol..

[34] Thøgersen J., Haugaard P., Olesen A. (2010). Consumer responses to ecolabels. Eur. J. Mark..

[35] Kiatkawsin K., Han H. (2017). Young travelers’ intention to behave pro-environmentally: Merging the value-belief-norm theory and the expectancy theory. Tour. Manag..

[36] Burlea-Schiopoiu A., Ogarca R.F., Barbu C.M., Craciun L., Baloi I.C., Mihai L.S. (2021). The impact of COVID-19 pandemic on food waste behaviour of young people. J. Clean. Prod..

[37] Przezbórska-Skobiej L., Wiza P.L. (2021). Food waste in households in Poland—Attitudes of young and older consumers towards the phenomenon of food waste as demonstrated by students and lecturers of PULS. Sustainability.

[38] Jokonya O. (2017). Critical Literature Review of Theory of Planned Behavior in the Information Systems Research. Proceedings of the 2nd International Conference on Advances in Management Engineering and Information Technology (AMEIT 2017).

[39] Solomon R.M., Bambossy J.G., Askegaard S., Hogg K.M. (2016). Consumer Behavior, A European Perspective, 6th edition.

[40] Jribi S., Ben Ismail H., Doggui D., Debbabi H. (2020). COVID-19 virus outbreak lockdown: What impacts on household food wastage?. Environ. Dev. Sustain..

[41] Aldaco R., Hoehn D., Laso J., Margallo M., Ruiz-Salmón J., Cristobal J., Kahhat R., Villanueva-Rey P., Bala A., Batlle-Bayer L. (2020). Food waste management during the COVID-19 outbreak: A holistic climate, economic and nutritional approach. Sci. Total Environ..

[42] Hebrok M., Boks C. (2017). Household food waste: Drivers and potential intervention points for design—An extensive review. J. Clean. Prod..

[43] Reynolds C., Goucher L., Quested T., Bromley S., Gillick S., Wells V.K., Evans D., Koh L., Carlsson Kanyama A., Katzeff C. (2019). Review: Consumption-stage food waste reduction interventions—What works and how to design better interventions. Food Policy.

[44] Whitehair K.J., Shanklin C.W., Brannon L.A. (2013). Written messages improve edible food waste behaviours in a university dining facility. J. Acad. Nutr. Diet..

[45] Martins L.M., Rodrigues S.S., Cunha L.M., Rocha A. (2015). Strategies to reduce plate waste in primary Schools—Experimental evaluation. Public Health Nutr..

[46] Stöckli S., Niklaus E., Dorn M. (2018). Call for testing interventions to prevent consumer food waste. Resour. Conserv. Recycl..

[47] Pinto R.S., dos Santos Pinto R.M., Melo F.F.S., Camps S.S., Cordovil C.M.D. (2018). A simple awareness campaign to promote food waste reduction in a University canteen. Waste Manag..

[48] Soma T., Li B., Maclaren V. (2020). Food Waste Reduction: A Test of Three Consumer Awareness Interventions. Sustainability.

[49] Baron S., Patterson A., Maull R., Warnaby G. (2018). Feed people first: A service ecosystem perspective on innovative food waste reduction. J. Serv. Res..

[50] Clement J., Aastrup J., Charlotte Forsberg S. (2015). Decisive visual saliency and consumers’ in-store decisions. J. Retail. Consum. Serv..

[51] Lim V., Funk M., Marcenaro L., Regazzoni C., Rauterberg M. (2017). Designing for action: An evaluation of Social Recipes in reducing food waste. Int. J. Hum. Comput. Stud..

[52] Thieme A., Comber R., Miebach J., Weeden J., Kraemer N., Lawson S., Olivier P. “We’ve bin watching you” designing for reflection and social persuasion to promote sustainable lifestyles. Proceedings of the SIGCHI Conference on Human Factors in Computing Systems (CHI ’12).

[53] von Kameke C., Fischer D. (2018). Preventing household food waste via nudging: An Exploration of consumer perceptions. J. Clean. Prod..

[54] Kallbekken S., Sælen H. (2013). ‘Nudging’ hotel guests to reduce food waste as a win-win environmental measure. Econ. Lett..

[55] Freedman M.R., Brochado C. (2010). Reducing Portion Size Reduces Food Intake and Plate Waste. Obes. Soc..

[56] National Academies of Sciences, Engineering, and Medicine (2020). A National Strategy to Reduce Food Waste at the Consumer Level.

[57] van der Werf P., Seabrook J.A., Gilliland J.A. (2021). “Reduce Food Waste, Save Money”: Testing a Novel Intervention to Reduce Household Food Waste. Environ. Behav..

[58] Quested T. (2019). Guidance for Evaluating Interventions Preventing Household Food Waste.

[59] Kvale S., Brinkmann S. (2009). InterViews: Learning the Craft of Qualitative Research Interviewing, 2nd Edition.

[60] Lorente A., Boersma K.F., Eskes H.J., Veefkind J.P., Van Geffen J.H.G.M., De Zeeuw M.B., Krol M.C. (2019). Quantification of nitrogen oxides emissions from build-up of pollution over Paris with TROPOMI. Sci. Rep..

[61] Marek-Andrzejewska E.M., Wielicka-Regulska A. (2021). Targeting Youths’ Intentions to Avoid Food Waste: Segmenting for Better Policymaking. Agriculture.

[62] Lithuanian Consumer Institute (2018). Sincerely, Food. https://www.vartotojai.lt/en/a-useful-little-book-about-food-waste-and-how-to-avoid-it-2018/#dearflip-df_8382/5/.

